# Synthesis of Anionic Phosphorus-Containing Heterocycles by Intramolecular Cyclizations Involving *N*-Functionalized Phosphinecarboxamides

**DOI:** 10.1002/chem.201500628

**Published:** 2015-03-03

**Authors:** Thomas P Robinson, Jose M Goicoechea

**Affiliations:** [a] Department of Chemistry, University of Oxford, Chemistry Research Laboratory12 Mansfield Road, Oxford, OX1 3TA (UK)

**Keywords:** 2-phosphaethynolate anion, heterocycles, phosphides, phosphorus, propargyl-phosphines

## Abstract

We report that the 2-phosphaethynolate anion (PCO^−^) reacts with propargylamines in the presence of a proton source to afford novel *N*-derivatized phosphinecarboxamides bearing alkyne functionalities. Deprotonation of these species gives rise to novel five- and six-membered anionic heterocycles resulting from intramolecular nucleophilic attack of the resulting phosphide at the alkyne functionality (via 5-*exo*-dig or 6-*endo*-dig cyclizations, respectively). The nature of the substituents on the phosphinecarboxamide can be used to influence the outcome of these reactions. This strategy represents a unique approach to phosphorus-containing heterocylic systems that are closely related to known organic molecules with interesting bio-active properties.

Phosphorus-containing heterocycles are molecules of appreciable interest, particularly due to their role as supporting ligands in homogeneous catalysis.[1,2] Nevertheless, the straightforward preparation of such compounds on a significant scale remains challenging, largely due to the fact that many of the chemical transformations available for the synthesis of more traditional “organic” heterocycles are not available to phosphorus-containing species. This is particularly true for systems with a variety of different functional groups where competing side-reactions may take place. One of the most recent developments in the synthesis of novel phosphorus–carbon heterocycles has been the use of the 2-phosphaethynolate anion, PCO^−^ (first reported by Becker and co-workers),[3,4] for the synthesis of novel four-, five- and six-membered ring systems.[3], 4, [5]–[8] In addition to affording molecules of fundamental importance, these recent studies have also produced redox-active systems which may ultimately be employed as components in electronic devices.[8]

We have recently developed an interest in designing phosphorus-containing molecules that may be used as precursors to novel heterocycles by exploiting existing paradigms for ring closure in organic compounds (or Baldwin’s rules).[9] This research builds on previous studies by our group demonstrating that PCO^−^ reacts with ammonium salts to afford phosphinecarboxamide, PH_2_C(O)NH_2_ (a heavier analogue of urea).[10] Herein we show that this reaction exhibits significant functional group tolerance for the synthesis of novel *N*-derivatized phosphinecarboxamides with reactive functional groups (in this case alkynes). Reaction of the 2-phosphaethynolate anion (PCO^−^) with propargylamines (NH_2_CH_2_CCH, NH_2_C(CH_3_)_2_CCH, and NH_2_CH_2_CC(C_6_H_5_)) in the presence of a proton source yields *N*-derivatized phosphinecarboxamides PH_2_C(O)NHCR_2_CCR′ (R=R′=H (**1**); R=CH_3_, R′=H (**2**); R=H, R′=C_6_H_5_ (**3**)) as pictured in Scheme 7.[11] These reactions proceed quantitatively and rapidly (by the time NMR data are collected on crude reaction mixtures). The resulting products can be identified by their ^31^P NMR spectra which reveal characteristic triplet resonances that collapse to singlets on proton decoupling (*δ*=−132.4, *δ*=−129.6, and *δ*=−132.6 ppm for **1**, **2**, and **3**, respectively, with ^1^*J*_H–P_ coupling constants of approximately 208 Hz). Full experimental details are provided in the Supporting Information. Compound **3** was found to be unstable in solution, decomposing to a mixture of unidentified compounds within an hour, although it can be generated in situ and used for subsequent transformations (vide infra). By contrast, **1** and **2** are indefinitely stable in solution and can be isolated in moderate to good yields as compositionally pure solids, as determined by field-ionization mass spectrometry and elemental analysis. Both were additionally characterized by single-crystal X-ray diffraction confirming the formation of *N*-(prop-2-yn-1-yl)phosphinecarboxamides (Figure 1 and Figure 2). To our knowledge, such compounds have not been reported previously, but are closely related to *N*-2-propyn-1-yl-acetamides (or *N*-propargylamides), which are commonly employed in organic chemistry, most notably for the generation 2,5-oxazoles.[12,13]

**scheme 1:**
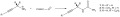
Formation of *N*-(prop-2-yn-1-yl)phosphinecarboxamides from the reaction of PCO^−^ with propargylamines in the presence of a proton source (pyridinium trifluromethansulfonate or HCl).

**Figure 1:**
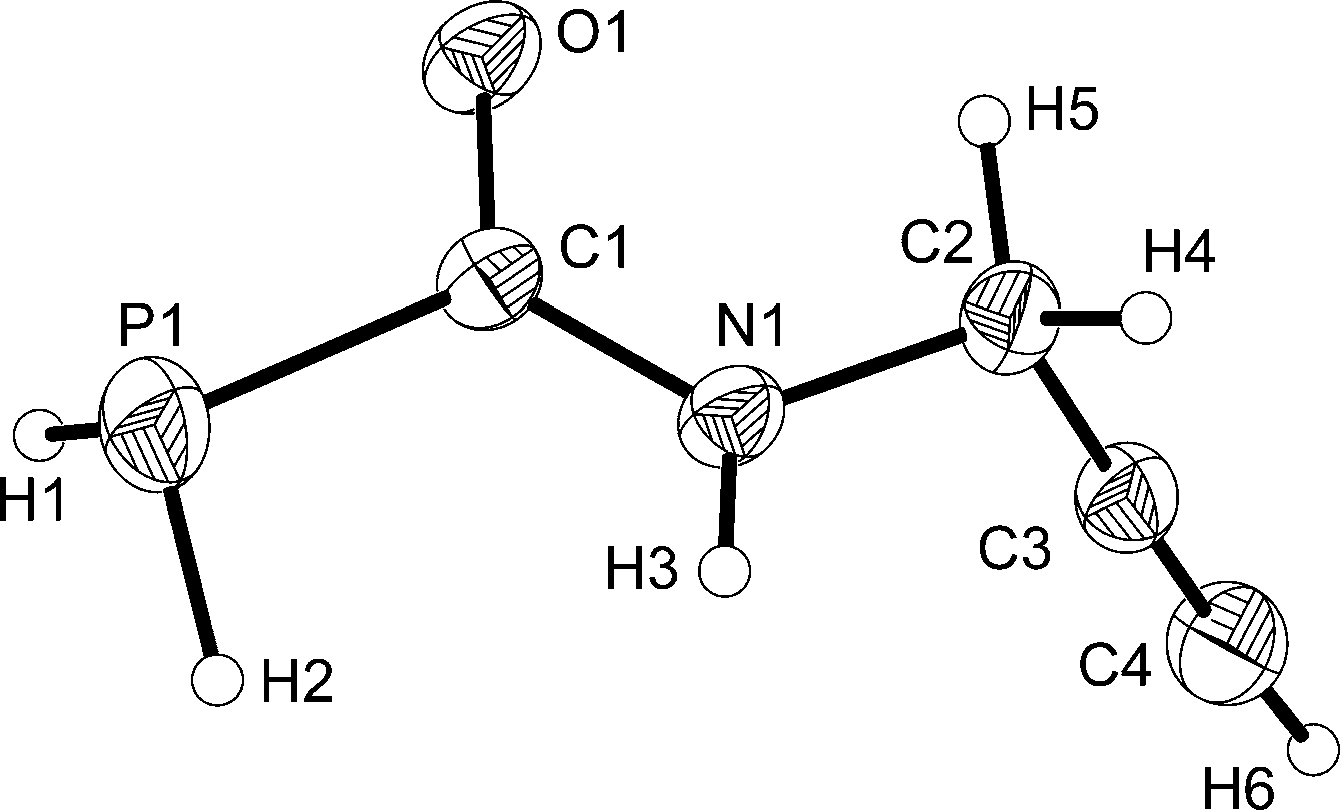
Molecular structure of 1. Anisotropic thermal displacement ellipsoids are pictured at the 50 % probability level. Hydrogen atom positions are pictured as spheres of arbitrary radius. Selected interatomic distances [Å] and angles [°]: P1—C1 1.860(1), P1—H1 1.15(3), P1—H2 1.24(3), C1—O1 1.240(2), C1—N1 1.332(2), N1—C2 1.460(2), N1—H3 0.85(2), C2—C3 1.464(2), C3—C4 1.188(2); P1-C1-O1 119.8(1), P1-C1-N1 117.0(1), O1-C1-N1 123.1(1).

**Figure 2:**
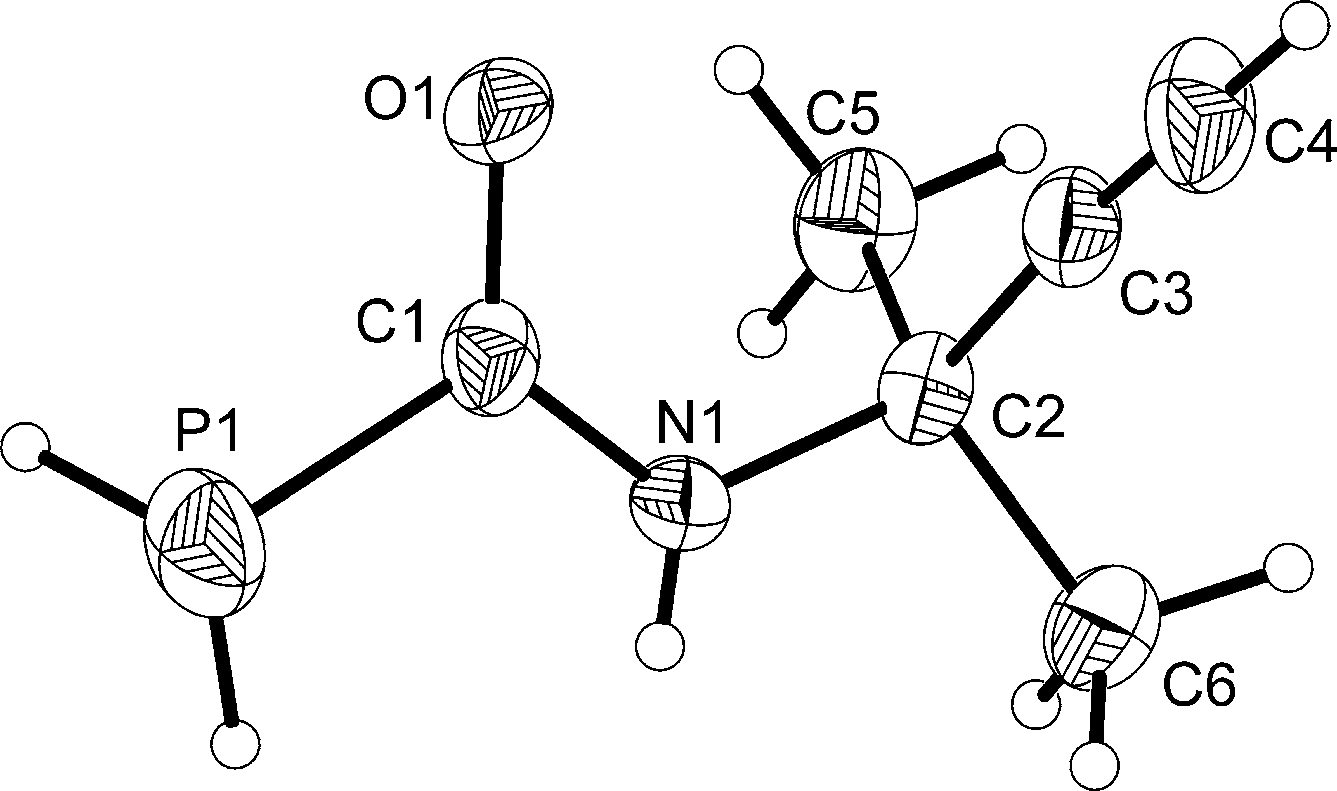
Molecular structure of 2. Anisotropic thermal displacement ellipsoids are pictured at the 50 % probability level. The molecule displays positional disorder in the lattice involving the PH_2_C(O)NH moiety. The minor component (5 % occupancy) has been removed for clarity. Hydrogen atom positions are pictured as spheres of arbitrary radius. Selected interatomic distances [Å] and angles [°]: P1—C1 1.862(2),, P1—H1 1.16(2), P2—H2 1.21(2), C1—O1 1.229(3), C1—N1 1.331(3), N1—C2 1.481(3), C2—C3 1.475(3), C2—C5 1.528(3), C2—C6 1.531(3), C3—C4 1.171(3); P1-C1-O1 118.3(2), P1-C1-N1 117.3(2), O1-C1-N1 124.3(3).

The structures of **1** and **2** exhibit comparable bond metrics and are closely related to that of PH_2_C(O)NH_2_. The P—C (**1**: 1.860(1) Å; **2**: 1.862(2) Å), C—O (**1**: 1.240(2) Å; **2**: 1.229(3) Å), and C—N distances (**1**: 1.332(2) Å; **2**: 1.331(3) Å) of the phosphinecarboxamide moiety are identical within experimental error and comparable to those recorded for PH_2_C(O)NH_2_ (P—C: 1.865(1) Å; C—O: 1.230(2) Å; C—N: 1.329(2) Å).[10] Moreover, both species also reveal the presence of a C—C triple bond with interatomic distances of 1.188(2) and 1.171(3) Å for **1** and **2**, respectively.

The optimized computed geometries at the density functional theory (DFT) level for **1**–**3** display closely related bond metric parameters, and those of **1** and **2** are in good agreement with the crystallographically determined values (see Supporting Information for further details).[14] The calculations show significant lone pair character on the phosphorus atom for the HOMO of **1** and **2** (42.39 % and 36.88 %, respectively) and the HOMO−1 of **3** (40.99 %), and large HOMO–LUMO gaps for all three species (ranging between 5.38 and 6.58 eV).

Deprotonation of **1** at −78 °C using one equivalent of KHMDS reveals a mixture of two products by ^31^P NMR spectroscopy at *δ*=−4.4 and −34.3 ppm. The former is a quartet resonance (^3^*J*_H-P_=8.5 Hz), whereas the latter appears as a doublet of doublets (^2^*J*_H–P_=41.0 Hz, ^3^*J*_H–P_=6.3 Hz)—both of which collapse to singlet resonances on proton decoupling. Based on these observations we were led to hypothesize that deprotonation of the phosphine moiety (-PH_2_) gives rise to an anionic phosphide that can attack the alkyne functionality in an intramolecular fashion. The outcome of such a process can give rise to two different products depending on whether the phosphide attacks the β or γ carbon (relative to the amide nitrogen). According to Baldwin’s rules, such reactions would either afford the 5-*exo*-dig or 6-*endo*-dig cyclization products, respectively (see Scheme 8). NMR spectroscopic data are consistent with the formation of [*cyclo*-PC(O)NHCHCH(CH_3_)]^−^ (**4**) and *cyclo*-[PC(O)NHCH_2_CHCH]^−^ (**5**) in a 1:4 ratio.

**scheme 2:**
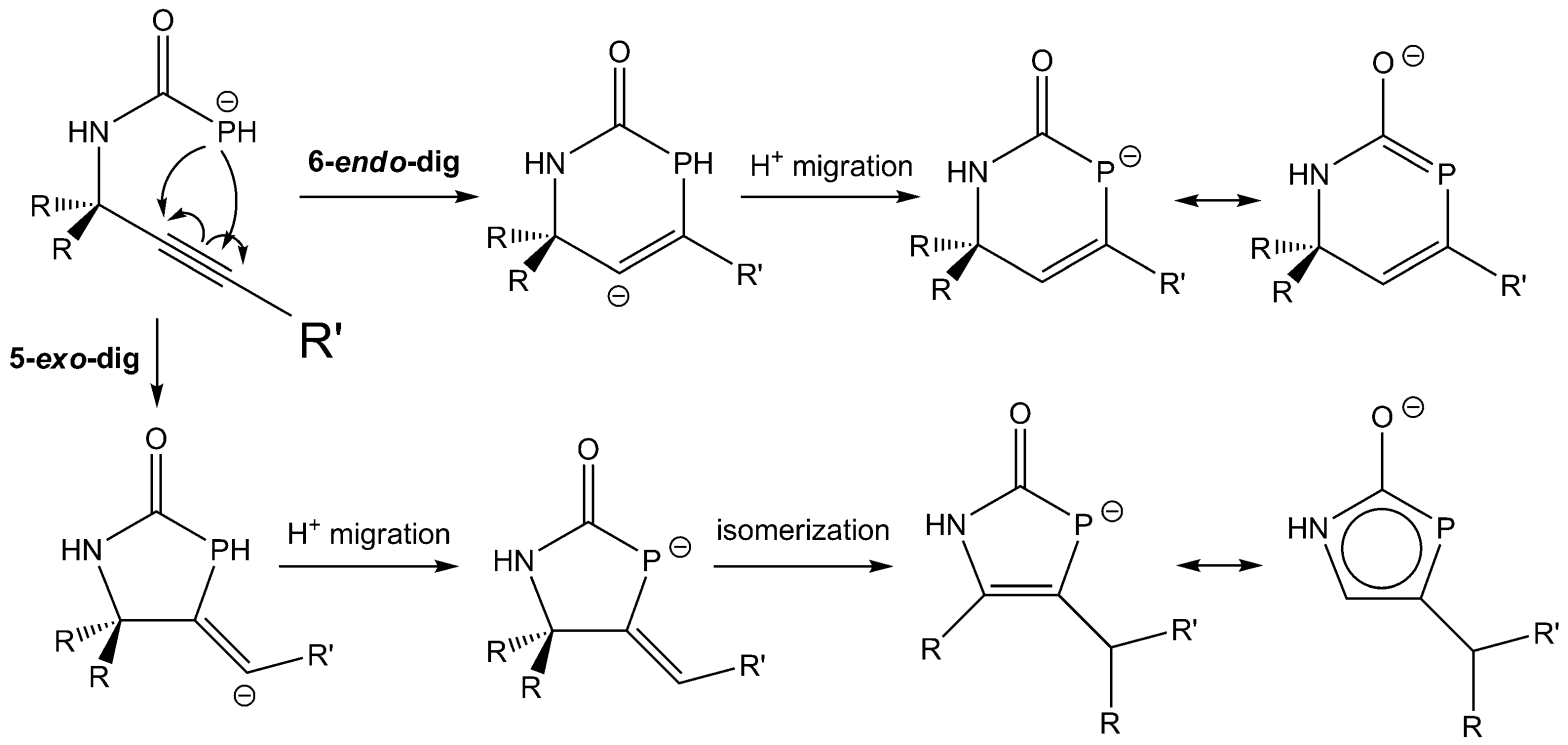
Possible cyclization reactions of deprotonated *N*-(prop-2-yn-1-yl)phosphinecarboxamides to afford the 6-*endo*-dig (top) and 5-*exo*-dig (bottom) products.

Due to their comparable solubility, the isolation of compositionally pure samples of **4** and **5** was not possible. Addition of 18-crown-6 (1,4,7,10,13,16-hexaoxacyclo-octadecane) to the reaction mixture allowed the crystallization of the 6-*endo*-dig product, **5**, which was crystallographically characterized as [K(18-crown-6)][**5**]⋅0.5 THF (Figure 3). The structure confirms the formation of a six-membered ring containing an intact carboxamide moiety. Bond metric data for this motif are consistent with significant delocalization of negative change between the phosphorus atom and the carbonyl moiety, observable in a shorter P1—C1 and longer C1—O1 distance (1.799(2) and 1.269(2) Å, respectively) relative to that of the *N*-(prop-2-yn-1-yl)phosphinecarboxamide precursor (P—C: 1.860(1) Å; C—O: 1.240(2) Å). The ring contains a C—C bond which is apparent in the C3—C4 distance (1.311(4) Å). This value is notably longer than the alkyne in **1** (1.188(2) Å).

**Figure 3:**
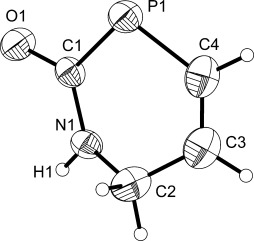
Molecular structure of the anionic moiety characterized in [K(18-crown-6)][5]⋅0.5THF. Anisotropic thermal displacement ellipsoids are pictured at the 50 % probability level. Hydrogen atom positions are pictured as spheres of arbitrary radius. Selected interatomic distances [Å] and angles [°]: P1—C1 1.799(2), P1—C4 1.786(2), C1—O1 1.269(2), C1—N1 1.353(3), N1—C2 1.450(3), C2—C3 1.483(4). C3—C4 1.311(4); P1-C1-O1 121.1(2), P1-C1-N1 119.4(1), O1-C1-N1 119.4(2), C1-N1-C2 121.8(2), N1-C2-C3 113.5(2), C2-C3-C4 118.3(2), C3-C4-P1 125.6(2), C4-P1-C1 99.6(1).

To our knowledge such six-membered phosphorus-containing heterocycles are entirely unprecedented. The structure is closely related to analogous cyclic urea systems such as 3,4-dihydropyrimidin-2(1 *H*)-ones. Such heterocyclic systems have attracted considerable interest in the organic chemistry community on account of their antiviral, antitumor, antibacterial, and anti-inflammatory activities.[15]

In an effort to selectively target compositionally pure samples of the aforementioned heterocycles, we attempted analogous cyclizations employing the functionalized *N*-(prop-2-yn-1-yl)phosphinecarboxamides PH_2_C(O)NHCR_2_CCR′ (R=CH_3_, R′=H (**2**); R=H, R′=C_6_H_5_ (**3**)). We postulated that introducing steric bulk at the α-carbon would favor the formation of the six-membered 6-*endo*-dig product. By contrast, aryl substituents on terminal alkynes have been demonstrated to favor formation of the 5-*exo*-dig products.[16]

Reaction of **2** with one equivalent of KHMDS in the presence of 18-crown-6 gives rise to a single product exhibiting a multiplet resonance in the ^31^P NMR spectrum at *δ*=−44.4 ppm (c.f. *δ*=−129.6 ppm for **2**). This doublet of doublets (^2^*J*_H–P_=40.4 Hz, ^3^*J*_H–P_=6.7 Hz) collapses to a singlet on proton decoupling. The downfield shift of this resonance compared to that of the phosphinecarboxamide precursor is consistent with the formation of a phosphide. The chemical shift and coupling constant data for this new species are closely related to those recorded for **5** and are consistent with the formation of the 6-*endo*-dig cyclization product [*cyclo*-PC(O)NHC(CH_3_)_2_CHCH]^−^ (**6**). Large colorless crystals of [K(18-crown-6)][**6**]⋅THF could be isolated in good yield by cooling a concentrated THF solution of the reaction product to −30 °C.

The single-crystal X-ray structure of [K(18-crown-6)][**6**]⋅THF (Figure 4) reveals a six-membered ring analogous to that of **5** (vide supra). As would be expected, bond metric data for both systems are very similar (P1—C1: 1.799(2) and 1.790(1); C1—O1: 1.269(2) Å and 1.258(1); C3—C4: 1.311(4) and 1.335(2), for **5** and **6**, respectively). The optimized computed geometries as determined by DFT analysis (using a continuum dielectric model to simulate solvation) were found to be in good agreement with experimentally determined values. Computed Hirshfeld [and Mulliken] charge values are consistent with a significant degree of negative charge delocalization over the phosphorus and oxygen atoms of the phosphinecarboxamide moiety (**5**: −0.336 and −0.470 [−0.318 and −0.613]; **6**: −0.331 and −0.474 [−0.307 and −0.619]). These values are in line with the observation that there is significant phosphorus and oxygen contribution to the highest occupied molecular orbitals.

**Figure 4:**
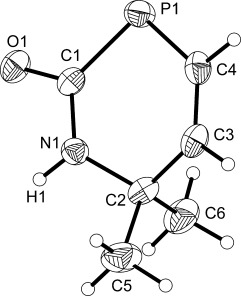
Molecular structure of the anionic moiety characterized in [K(18-crown-6)][6]⋅THF. Anisotropic thermal displacement ellipsoids are pictured at the 50 % probability level. Hydrogen atom positions are pictured as spheres of arbitrary radius. Selected interatomic distances [Å] and angles [°]: P1—C1 1.790(1), P1—C4 1.797(1), C1—O1 1.258(1), C1—N1 1.380(1), N1—C2 1.471(1), C2—C3 1.507(2), C2—C5 1.527(2), C2—C6 1.534(2), C3—C4 1.335(2); P1-C1-O1 121.7(1), P1-C1-N1 120.9(1), O1-C1-N1 117.2(1), C1-N1-C2 125.2(1), N1-C2-C3 110.1(1), C5-C2-C6 110.3(1), C2-C3-C4 123.2(1), C3-C4-P1 127.1(1), C4-P1-C1 99.0(1).

The in situ generation of **3**, and subsequent reaction with KHMDS in the presence of 18-crown-6 gives rise to a single reaction product exhibiting a multiplet resonance at *δ*=−16.2 ppm (dt, ^3^*J*_H–P_=13.4 Hz, ^3^*J*_H–P_=4.5 Hz). This species was crystallized from THF at −30 °C and identified as a five-membered ring containing an *exo*-double bond [*cyclo*-PC(O)NHCH_2_C{CH(C_6_H_5_)}]^−^ (**7**; Figure 5).

**Figure 5:**
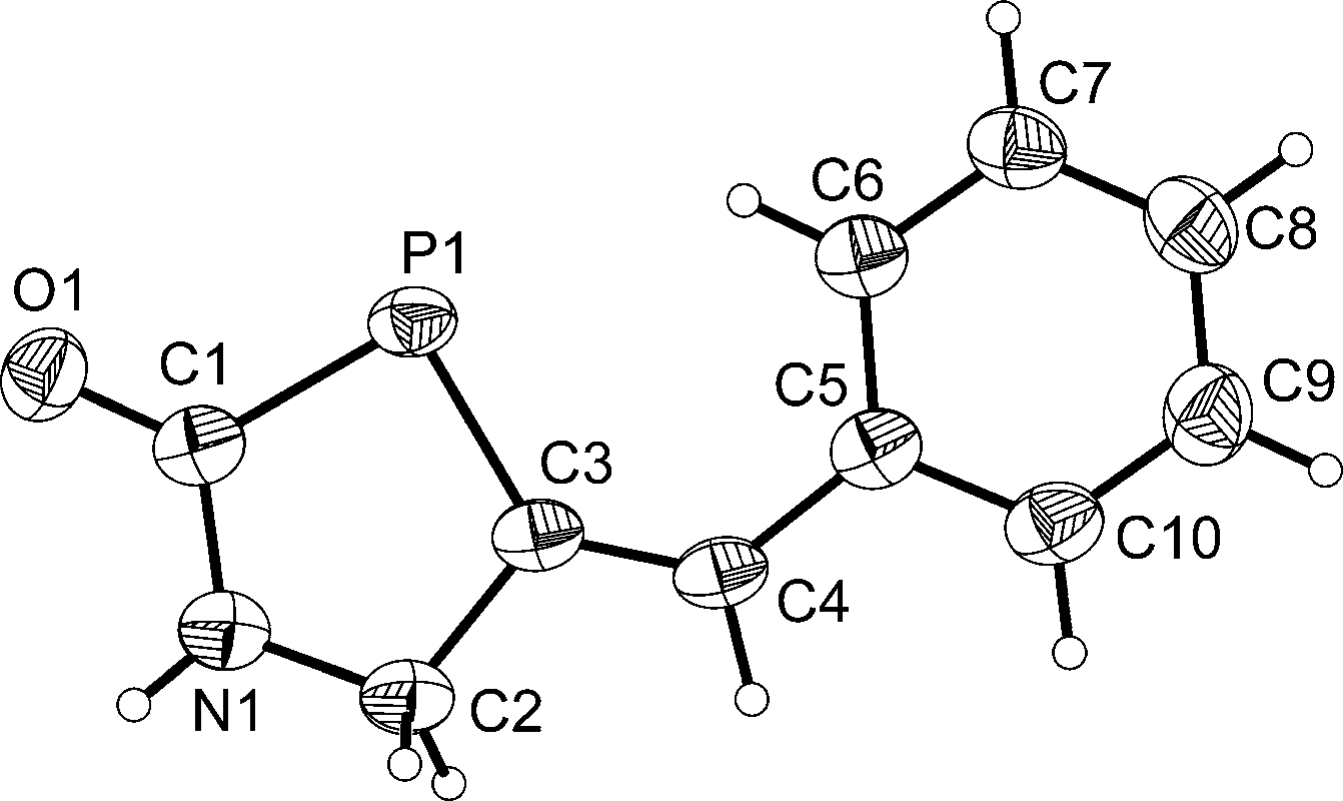
Molecular structure of the anionic moiety characterized in [K(18-crown-6)][7]. Anisotropic thermal displacement ellipsoids are pictured at the 50 % probability level. Hydrogen atom positions are pictured as spheres of arbitrary radius. Selected interatomic distances [Å] and angles [°]: P1—C1 1.817(2), P1—C3 1.798(2), C1—O1 1.249(3), C1—N1 1.362(3), N1—C2 1.448(3), C2—C3 1.518(3), C3—C4 1.359(3), C4—C5 1.456(3), C5—C6 1.404(3), C5—C10 1.409(3), C6—C7 1.394(3), C7—C8 1.385(4), C8—C9 1.388(4), C9—C10 1.386(4); P1-C1-O1 125.8(2), P1-C1-N1 112.7(2), O1-C1-N1 121.5(2), C1-N1-C2 116.0(2), N1-C2-C3 109.2(2), C2-C3-P1 110.6(2), C2-C3-C4 117.5(2), C3-C4-C5 129.9(2), P1-C3-C4 132.0(2).

On heating solutions of **7**, isomerization of the heterocycle is observed involving proton transfer from the methylene moiety of **7** to the *exo*-double bond affording [*cyclo*-PC(O)NHCHC{CH_2_(C_6_H_5_)}]^−^ (**8**). This finding is consistent with theoretical calculations which show that isomer **8** is −9.9 kJ mol^−1^ more stable than **7**. The isomerization is accompanied by a shift of the ^31^P NMR resonance from *δ*=−16.2 to −3.5 ppm (this value is closely related to that recorded for **4** which was observed at *δ*=−4.4 ppm). Crystals of [K(18-crown-6)][**8**]⋅1.5 py suitable for single-crystal X-ray diffraction were grown by diffusion of hexane into a concentrated pyridine solution of the product (Figure 6). On account of the similarity of both isomers, bond metric details are discussed in tandem.

**Figure 6:**
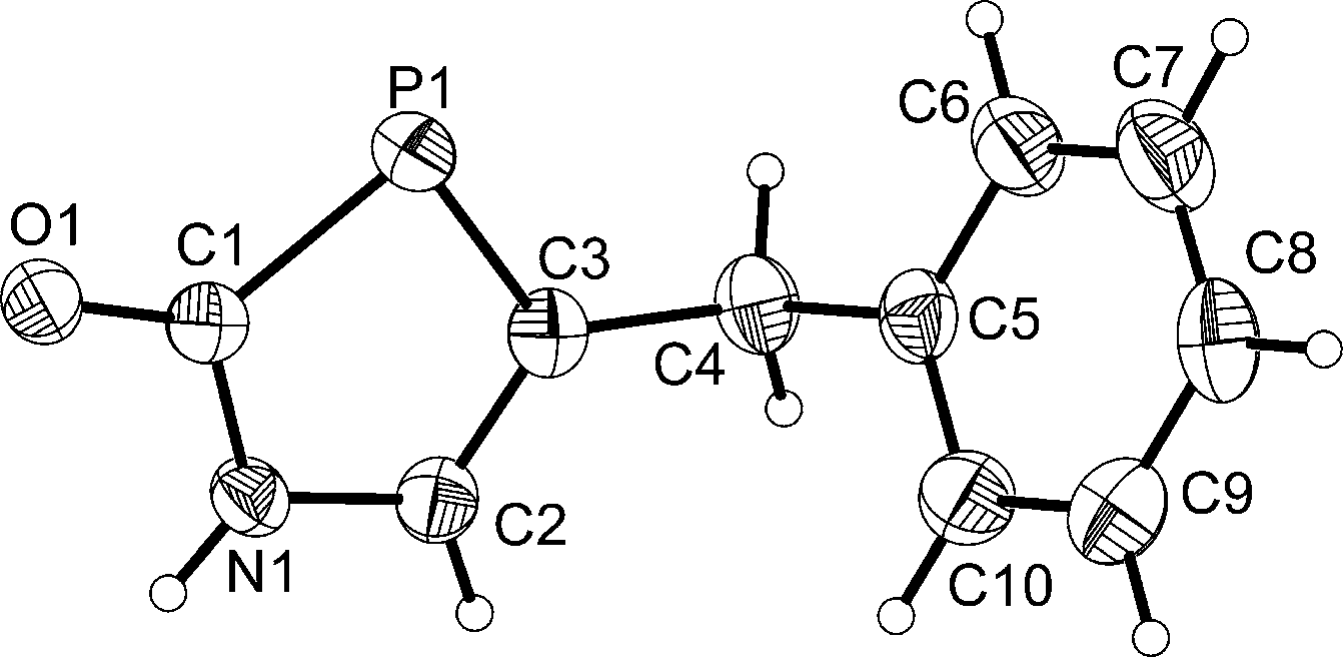
Molecular structure of the anionic moiety characterized in [K(18-crown-6)][8]⋅1.5py. Anisotropic thermal displacement ellipsoids are pictured at the 50 % probability level. Hydrogen atom positions are pictured as spheres of arbitrary radius. Selected interatomic distances [Å] and angles [°]: P1—C1 1.788(2), P1—C3 1.791(2), C1—O1 1.277(2), C1—N1 1.376(2), N1—C2 1.382(3), C2—C3 1.350(3), C3—C4 1.504(3), C4—C5 1.514(3), C5—C6 1.385(3), C5—C10 1.384(3), C6—C7 1.382(3), C7—C8 1.382(4), C8—C9 1.378(4), C9—C10 1.387(3); P1-C1-O1 128.5(1), P1-C1-N1 110.3(1), O1-C1-N1 121.2(2), C1-N1-C2 114.6(2), N1-C2-C3 114.5(2), C2-C3-P1 111.4(2), C2-C3-C4 123.8(2), C3-C4-C5 113.3(2), P1-C3-C4 124.8(2).

Both crystal structures contain a five-membered anionic ring system with a phosphine carboxamide moiety. Interatomic distances for the P—C, C—O, and C—N bonds of the phosphinecarboxamide core are very similar in both systems (P—C: 1.817(2) and 1.788(2) Å; C—O: 1.249(3) and 1.277(2) Å; C—N: 1.362(3) and 1.376(2) Å, for **7** and **8**, respectively). The most notable structural variations involve the *exo*-substituent, in the case of **7**, the C3—C4 bond length is consistent with double bond character 1.359(3) Å. By contrast, in **8** the bond lengthens significantly to 1.504(3) Å; this is accompanied by a concomitant shortening of the N1—C2 bond to 1.382(3) Å (c.f. 1.448(3) Å in **7**) and of the C2—C3 bond to 1.350(3) Å (1.518(3) Å in **7**). Both anions are related to *P*-derivatized 1,3-azaphosphol-2-ones, previously reported in the chemical literature.[17] As with **5** and **6**, the optimized computed geometries from the DFT analysis are in close agreement with the experimentally determined ones. In both cases, there is a significant distribution of negative charge between the phosphorus and oxygen atoms of the heterocycle, with a greater accumulation of charge on the oxygen atoms in both isomers.

We have demonstrated that *N*-functionalized phosphinecarboxamides (PH_2_C(O)NHR) bearing reactive functional groups are available using the phosphorus-containing analogue of the cyanate anion (PCO^−^). Intramolecular transformations involving these species allow the synthesis of unprecedented five- and six-membered heterocycles. In addition to their fundamental interest, these novel compounds may ultimately be used for the synthesis of chiral phosphines and as precursors to polymeric materials. Studies on the subsequent reactivity of **1**–**8** are currently on-going.
